# Applying multi-theory model to determine intentions to smoking cessation among male health worker smokers in Kabul, Afghanistan

**DOI:** 10.3389/fpubh.2024.1472498

**Published:** 2025-01-08

**Authors:** Mousa Bashir, Farkhondeh Amin Shokravi, Anoshirvan Kazemnejad

**Affiliations:** ^1^Department of Health Education and Health Promotion, Faculty of Medical Sciences, Tarbiat Modares University, Tehran, Iran; ^2^Department of Biostatistics, Faculty of Medical Sciences, Tarbiat Modares University, Tehran, Iran

**Keywords:** smoking cessation, multi-theory model, initiation, sustenance, health workers

## Abstract

**Introduction:**

Smoking causes lung cancer and a wide range of acute and chronic diseases annually throughout the world. A fourth-generation behavioral framework, namely the Multi-Theory Model (MTM) of health behavior change was used to predict the initiation and maintenance of smoking cessation among health worker smokers.

**Methods:**

A cross-sectional study of 170 smoking healthcare workers was conducted in Kabul. A non-probability convenience sampling method was used to recruit respondents. A valid and reliable 37-item MTM-based questionnaire was administered to male smokers. Stepwise multiple regression was used to explain smoking cessation. The overall Cronbach’s alpha coefficient (*α*) for the initial and retention scales of the MTM variables was 0.80 and 0.79, respectively.

**Results:**

The average age of the participants was 29.33 ± 6.21 years. The reported average year of smoking was 5.6 ± 4.7 with the average number of 5.64 ± 5.21cigarette smoking per day. Behavioral confidence and changes in physical environment were significant predictors of smoking cessation initiation. The sustenance of smoking cessation behavior was significantly influenced by emotional transformation, practicing for change and changes in social environment.

**Conclusion:**

MTM has the usefulness to assess both the initiation and sustenance behavior of smoking cessation. Potential arrangements utilizing MTM develops ought to be created in future interventions to alter behavior of smoking cessation.

## Introduction

Smoking is one of the leading causes of preventable deaths worldwide (1). Based on the report of WHO in 2024, trend of tobacco use among men on a global scale has been declining since 2000. Starting from 40.1% in 2000, it has decreased to 35.5% in 2020, 34.4% in 2022, is expected to reach 32.9% in 2025 and is projected to 30.2% in 2030 (2). Also, the study ‘Evolution of the global smoking epidemic over the past half century’ in 2022 demonstrates that the global male smoking rate is 32.6% (3). The prevalence of smoking in men aged ≥15 years in 2020 in some Middle East region and neighboring countries was as follows: Pakistan is 35, Iran is 20, and India is 17.8% (4). The WHO reported the prevalence of smoking in Afghan men aged ≥15 years to be 35% in 2017. The report added that out of the five most common cancers among Afghan men, four cancers (stomach, esophagus, lung, and oral cavity) are attributed to smoking and other forms of tobacco use (5). Smoking damages almost all organs of the body, leads to countless diseases, and reduces the level of health in smokers (6). According to Malaina et al., cardiovascular diseases (CVDs) are linked to smoking, and health outcomes can be improved by changing this behavior (7). To change such a behavior, especially in the process of smoking cessation, health workers can play an essential role because they are both counselors and smoking cessation models for citizens (8). Training programs are needed to increase their ability to actively support patients through smoking cessation techniques (9, 10). Studies show that if health workers are not smokers themselves, they will be more successful in persuading others to quit smoking. They will also be more supported and counseled by healthcare (11). In addition, there is a relatively high rate of smoking among health workers, especially in some countries like Iraq (26.5%) (12); however, they receive little formal training to quit smoking (7.3%) (13). Since they usually have little knowledge about smoking cessation strategies (14), so they are not good role models for their patients (15). It has been shown that although public health interventions to promote smoking cessation are effective, but the potential for applying such interventions across community is limited. There is inadequate predictive power in previous health behavior models used to describe smoking cessation, and long-term behavioral change cannot be evaluated using those models (16). As a health behavior theory, the Multi-Theory Model (MTM) employs a fourth-generation framework for simultaneous prediction and modification of health behaviors over a long period of time (17, 18). It is an efficient theory for assessing both the initiation and sustenance of smoking prevention, reduction, and cessation (16, 19–22). Moreover, health behaviors have been explained in a wide variety of populations using the MTM (23–33).

**Figure 1:**
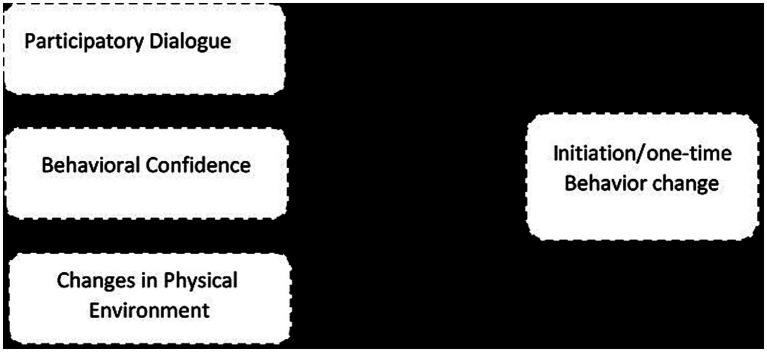
Constructs of smoking cessation initiation in the multi-theory model (MTM).

As shown in Figure 1, initiation of a behavior model has three constructs:

(1) Participatory dialog (advantages and the disadvantages of the health behavior change).
(2) Behavioral confidence (beliefs that one can perform the behavior change).
(3) Changes in physical environment (having resources at one’s disposal for behavior change) (34, 35).

**Figure 2:**
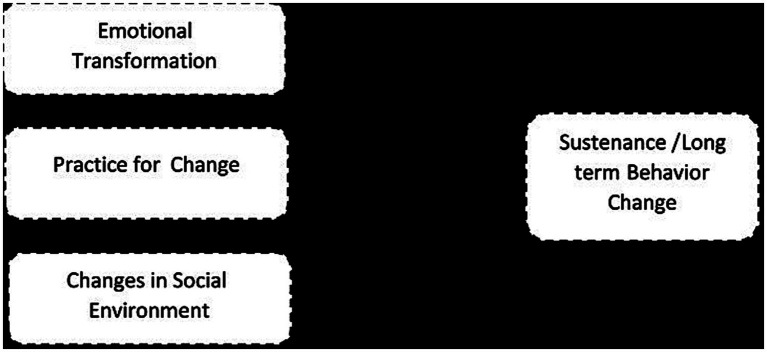
Constructs of smoking cessation sustenance in MTM.

Constructs can be divided into three types to maintain behavior (See Figure 2):

(1) Emotional transformation (translating feelings into goals for behavior change).
(2) Practice for change (creating a habit of transformation and making it a way of life).
(3) Changes in social environment (obtaining social support to help one maintain the health behavior change) (34, 35).

Since there are few studies about tobacco in Afghanistan, we applied the MTM for determining intentions toward smoking cessation among Afghan male health worker smokers.

## Materials and methods

The present cross-sectional research was conducted in the west of Kabul City (Afghanistan) on male health worker smokers within April–July 2021. The inclusion criteria were smoking one or more cigarettes during the past 7 days and having ≥20 years of age. The sample size was calculated according to the Cochran’s formula (95% confidence and 3% error), and the number of samples for the descriptive phase was obtained as follows:


$$n=\frac{Z_{1-\frac{\alpha }{2}}^{2}p\left(1-p\right)}{d^{2}}=\frac{1.96\times 0.265\times 0.735}{0.03^{2}}=\frac{0.382}{0.0009}=424.4≅425$$

“n” is the sample size, for a 95% confidence interval, “Z₁-*α*/₂” = 1.96, “p” = estimated proportion of the population (in this case, *p* = 0.265, the estimated proportion of smokers in the population), the percentage of non-smokers is represented by “1 – p” = 0.735 (1–0.265 = 0.735), “d”: the margin of error (set to 0.03, representing a 3% margin of error).

As shown, it is equal to 425 people, which is equal to 595 people considering 40% drop. It needs to be randomly selected and we expect at least 170 smokers in the sample to be investigated. Due to the cost and feasibility during the research period, convenience sampling was employed by visiting health centers to select participants. Healthcare workers, who were easily available through visits to nearby health centers, completed the questionnaire. To ensure data quality, several measures were put in place. First, all participants were informed about the study’s purpose and provided their informed consent before taking part. Data were collected through face-to-face interviews at health centers to ensure clarity and minimize misunderstandings. The researcher was present during the data collection process to assist participants with completing the questionnaires and to ensure consistency and accuracy. Any incomplete or inconsistent responses were carefully reviewed with the participants, and problematic data were excluded from the analysis to maintain integrity.

For Stepwise multiple regression, a minimum sample size of 170 participants is required if the alpha is 0.05, the power is 0.90, effect size is equal to 0.1, and response rate is 80. Each model includes three predictors and five covariates using G power 3.1 software (36).

The independent variables were initiation model components (participatory dialog, behavioral confidence, and changes in physical environment) and the sustenance model components (emotional transformation, practice for change, and changes in social environment). The dependent variables in both models were intention to initiate and sustain smoking cessation behavior changes.

### Instruments

In this study, a self-report MTM-based questionnaire consisting of 37 items was used (37). The first eight questions assess a person’s smoking status (number of cigarettes smoked per day, years of smoking, and current smoking status), and demographic characteristics (education level, age, income, and employment status). The remaining 29 items are used to assess the MTM constructs associated with the two models: (a) Initiation model, including participatory dialog, behavioral confidence, and changes in physical environment, and (b) Sustenance model, including emotional transformation, practice for change, and changes in social environment (16).

The original questionnaire was translated into Persian and then re-translated into English. Two translators, under the researcher’s supervision, translated the questionnaire to Persian, and then two other translators retranslated it into English. A meeting was held to review and adjust the translations, identify and resolve inappropriate phrases or concepts, and examine possible discrepancies between the original and translated versions. This group was responsible for combining the translations and creating a single version. The initial translated versions were compared, differences and inconsistencies were corrected, and the final translation in the target language was obtained by integrating the initial translations. Participants in this group were fluent in both the reference and target languages. The backward translation process involved two experts fluent in both the reference and target languages independently, ensuring that the final translated version was returned to the original language. The translators were not involved in the previous stages of the translation process, as it was done conceptually rather than word by word. The translated versions or translations made by the coordinator (researcher) were reviewed by the same previous group and discussed regarding contradictions and differences to reach a consensus. Finally, the translated version was sent to the questionnaire designer and approved by him.

### Validity and reliability

In the present study, a questionnaire developed in 2017 by Professor Sharma et al., entitled *“Quitting smoking by applying a new theory: Change in behavior using multi-theory models,”* was used. Its validity and reliability have been measured and established (37). The questionnaire was pilot tested with 30 health workers to determine its face validity. For this purpose, it was reviewed whether the items were easy to understand, clear, and straightforward. To verify face validity, several participants were interviewed face-to-face. Cronbach’s alpha (*α*) values of 0.80 and 0.79 were, respectively, used to establish internal consistency reliability for the subscales of initiation and sustenance of MTM variables.

Demographic Information: It contains information on age (years), Work per week (hr), How long smoke (year), How many cigarettes (daily), Income (1,000 AF), Degree and Marital status.

### Smoking cessation: initiation and sustenance

It is important to understand that initiation of smoking cessation includes deciding to quit smoking, while sustenance relates to the achievement of abstinence (37). In both items, the following ratings are given: not at all likely (0), somewhat likely (1), moderately likely (2), very likely (3), and completely likely (4) (37).

The initiation model is comprised of three constructs that are measured by 19 items:

(a) *Participatory dialog* that provides information on how to quit smoking and its advantages and disadvantages. Smoking cessation benefits are discussed in five questions in advantages (i.e., being healthy, able to save money, getting sick less often, smelling better, and enjoy life more) and five questions in disadvantages related to the difficulties of smoking cessation (i.e., not able to relax, not able to socialize, miss it, not able to overcome the urge and lose friends). The advantages and disadvantages were rated as: *never (1), almost never (2), sometimes (3), fairly often (3)*, and *very often (4)*. A possible total score ranged from 0 to 20. According to this hypothesis, higher scores for advantages and lower scores for disadvantages were related to the initiation of smoking cessation or behavior change. By subtracting disadvantages from advantages, the participatory dialog score was obtained, which ranged from −20 to +20 (13, 14).
(b) *Behavioral confidence*, a five-item assessment, which is used to determine whether a person is confident about quitting smoking (i.e., ability to quit smoking this week; ability to quit smoking this week and complete all work-related tasks; ability to quit smoking this week and feel relaxed, ability to quit this week without getting anxious; and ability to quit smoking this week without getting Withdrawal symptoms). In each item, the responses were rated as follows: *not sure at all (0), slightly sure (1), moderately sure (2), very sure (3)*, and *completely sure (4)*. The possible total scores ranged from 0 to 20. Higher scores imply more likelihood to initiate smoking cessation (37).
(c) Changes in *Physical environment* with three questions that refers to surrounding environment (i.e., ability to get rid of all cigarettes this week, ability not to buy cigarettes this week, and ability to substitute smoking time with something else this week). The response to each item was rated as follows: *not sure at all (0) slightly sure (1), moderately sure (2), very sure (3)*, and *completely sure (4)*. The possible total score range was 0–12. High-scoring individuals are more likely to initiate a smoking cessation program (37).

There were three constructs used to assess the constructs of the sustenance model:

(a) *Emotional transformation*, which consists of three questions related to emotions providing smoking cessation assistance, i.e., the ability to direct one’s emotions/feelings to the goal of being smoke-free every week, the ability to motivate oneself to be smoke-free every week, and ability to overcome self-doubt in accomplishing the goal of being smoke-free. The following grading system was used for each item: *not sure at all* (0) *slightly sure (1), moderately sure (2), very sure (3)*, and *completely sure* (4). In total, 12 scores were possible. A higher score indicates a greater likelihood of sustained quitting (37).
(b) *Practice for changes* with three items by which you can quit smoking (i.e., ability to keep a weekly self-diary to monitor smoking urges when faced with barriers, be smoke-free every week, and change the plan to be smoke-free every week if problems arise). Responses to each item were rated as follows: *from not at all sure (0)* to *completely sure (4)*. The range of possible scores was 0–12. Higher scores imply more likeliness to sustain their smoking cessation efforts (37).
(c) *Changes in social environment* with three items to determine whether family members, friends, and health care providers are likely to support you (i.e., ability to get weekly support from a family member to quit smoking, ability to get assistance from a friend each week, try not to smoke, and ability to receive assistance from a health professional to quit smoking every week). Responses to each item were rated as follows: *from not at all sure (0)* to *completely sure (4)*. The possible total scores ranged from 0 to 12. Higher scores indicate a greater chance of success in quitting smoking (37).

### Ethical aspects

The Research Ethics Committee of Tarbiat Modares University granted ethics approval for this study (IR.MODARES.REC.1399.256). The participants were initially informed of the research objectives, and all phases of the process were conducted in strict confidentiality. We collected no personal information from the participants and the participation was completely voluntary. A written informed consent was obtained from all participants, and the correct information was requested.

### Statistical analysis

The data gathered underwent analysis through IBM SPSS 26. Categorical variables are presented as count and percentage; continuous variables are presented as mean ± standard deviation. A stepwise regression analysis was carried out to ascertain the optimal predictors for changes in smoking cessation behavior, specifically in terms of initiation and maintenance, with demographic attributes being considered. The level of significance was set at *p* > 0.05. The outcome section includes the presentation of the scale factor for both the total and subscale variables. To provide further clarity on the statistical results:

**Cronbach’s Alpha** is used to assess how consistently a set of items measures a single concept. The coefficient ranges from 0 to 1, with higher values indicating better reliability. A Cronbach’s Alpha of 0.7 or above is generally considered acceptable for good internal consistency. Values above 0.8 suggest strong reliability, and values above 0.9 indicate excellent reliability. However, very high values may indicate redundancy among the items. On the other hand, values below 0.7 may suggest lower reliability of the scale.

**Standard Error (SE)**: This measure shows how much the “unstandardized coefficient” (B) might vary across different samples. In simple terms, it tells us how precise our estimate of the coefficient is. A smaller SE means the estimate is more reliable.

**t-Statistic (t)**: The t-statistic is calculated by dividing the “unstandardized coefficient” (B) by its standard error (SE). It helps us determine whether the coefficient is significantly different from zero. A higher t-value suggests that the predictor has a stronger, more meaningful relationship with the dependent variable.

**Unstandardized Coefficients** (B): These coefficients represent the change in the log-odds of the dependent variable (smoking cessation initiation) for a one-unit increase in the predictor variable, while keeping other predictors constant. For example, the unstandardized coefficient for Behavioral Confidence is 0.054, meaning that for each unit increase in behavioral confidence, the log-odds of initiating smoking cessation increase by 0.054.

**Standardized Coefficients** (Beta): These coefficients represent the strength of the relationship between the predictors and the dependent variable, scaled in terms of standard deviations. A higher Beta indicates a stronger effect. For instance, Changes in Physical Environment has a Beta of 0.355, indicating that it is the most influential predictor in the model.

**Adjusted R**^**2**^ is a modified version of R^2^ that adjusts for the number of predictors in the model. It provides a more accurate measure of model fit by penalizing excessive use of predictors that do not improve the model. The adjusted R^2^ value of 0.289 suggests that the predictors included in the model explain about 28.9% of the variance in smoking cessation initiation behavior, accounting for the number of predictors. The R^2^ value of 0.299 indicates the overall explanatory power of the model without adjusting for the number of predictors.

## Results

Participants meeting the inclusion criteria (aged 20 and over and smoked one or more cigarettes during the past 7 days) totaled 170 and filled out the questionnaire on paper. Table 1 provides a comprehensive Socio-demographic characteristics information of the participants. The mean age of the participants was 29.33 ± 6.21 years. According to the participants, the average number of hours worked per week was 39.88 ± 27.26 and they smoked for an average of 5.6 ± 4.7 years, with the average number of 5.64 ± 5.21 cigarettes per day. Table 2 presents a detailed overview of the Descriptive statistics of the participants’ socio-demographic characteristics, including income, education level, and marital status. These factors provide important context for understanding the profile of our study sample. The participants typically had an income between 20,000 and 50,000 AF, 47.6% of the participants had a bachelor’s degree, and 58.9% were married. Table 3 describes all MTM constructs together with their descriptive statistics and reliability calculations. Based on a 5-point Likert scale, there was a mean score of 2.19 ± 1.26 for the intention of initiation. In the initiation model, participatory dialog had a mean of 4.15 ± 4.96 units, behavioral confidence had a mean of 9.10 ± 5.41 units, and changes in physical environment had a mean of 6.01 ± 2.97. Moreover, there was a mean score of 2.08 ± 1.21 for the intention of sustenance. In the sustenance model, emotional transformation had a mean of 6.62 ± 3.00, practice for change had a mean of 6.01 ± 3.06, and changes in social environment had a mean of 7.02 ± 2.80. Table 4 reveals a strong and statistically significant relationship between Initiation and both Changes in Physical Environment and Behavioral Confidence. In contrast, no significant correlation was observed between Initiation and Participatory Dialog. On the other hand, Sustenance is positively and significantly correlated with Emotional Transformation, Practice for Change, and Changes in Social Environment. A Stepwise multiple regression was utilized to pinpoint predictors indicating that behavioral confidence and changes in physical environment were significant predictors of initiating smoking cessation (ADJ.R^2^ = 0.289). Emotional transformation, practicing for change, and changes in social environment significantly affected the maintenance of smoking cessation behavior (ADJ.R^2^ = 0.323). Stepwise multiple regression analysis displayed in Tables 5, 6. Independent variable of changes in physical environment is the most important predictor (26.9%). In the second step, the independent variable of behavioral confidence was added to the model, the predictive power of the variance related to the initiation for smoking cessation reached 28.9%. The independent variable of Emotional Transformation is the most important predictor (25.3%). In the second step, the independent variable of Practice for Change was added to the model. The predictive power of the variance related to the sustenance for smoking cessation reached 29.5%. In the third step, the independent variable of Changes in Social Environment was added to the model, and the predictive power of the variance related to Sustenance for smoking cessation reached 32.3%.

**Table 1:** Socio-demographic characteristics of the participants.

Variables	Mean ± SD
Age	29.33 ± 6.21
Work per week (hour)	39.88 ± 27.26
How long smoke (year)	5.6629 ± 4.72
How many cigarette (daily)	5.64 ± 5.21

**Table 2:** Descriptive statistics of the participants’ socio-demographic characteristics.

Variables		Frequency (Percentage)
Income (1,000 Af)	Less than 10	38(23.2%)
	10 to 20	51(31.1%)
	20 to 50	56(34.1%)
	50 to 100	15(9.1%)
	More than 100	4(2.4%)
Degree	Associate degree	37(22%)
	Bachelor’s degree	80(47.6%)
	Master’s degree or higher	51(30.4%)
Marital status	Married Single	99(58.9%) 69(41.1%)

**Table 3:** Descriptive statistics of the MTM constructs (*n* = 170).

Constructs	Possible range	Observed range	Mean ± SD	Cronbach’s alpha
Initiation	0 to 4	0 to 4	2.19 ± 1.26	0.804
Participatory dialog	−20 to 20	−10 to 14	4.15 ± 4.96	
Behavioral confidence	0 to 20	0 to 20	9.10 ± 5.41	
Changes in physical environment	0 to 12	0 to 12	6.01 ± 2.97	
Sustenance	0 to 4	0 to 4	2.08 ± 1.21	0.795
Emotional transformation	0 to 12	0 to 12	6.62 ± 3.00	
Practice for change	0 to 12	0 to 12	6.01 ± 3.06	
Changes in social environment	0 to 12	0 to 12	7.02 ± 2.80	

MTM, multi-theory model.

**Table 4:** Pearson’s correlation coefficient between the MTM constructs.

	1	2	3	4	5	6	7	8
1. Participatory Dialog	1							
2. Behavioral Confidence	−0.031	1						
3. Changes in Physical Environment	−0.125	0.723**	1					
4. Initiation	**−0.055**	0.520**	0.523**	1				
5. Emotional Transformation	0.036	0.756**	0.730**	0.561**	1			
6. Practice for Change	−0.163*	0.561**	0.596**	0.561**	0.616**	1		
7. Changes in Social Environment	0.021	0.220**	0.318**	0.561**	0.271**	0.265**	1	
8. Sustenance	−0.225**	0.490**	0.461**	0.561**	0.506**	0.473**	0.274**	1

*Correlation is significant at the 0.05 level. **Correlation is significant at the 0.01 level. Based on the table above, the intention to start smoking cessation showed a significant positive correlation with Behavioral Confidence and changes in Physical Environment (P < 0.0001), while it did not correlate with Participatory Dialogue (P = 0.496). Additionally, the Pearson correlation test revealed that the maintenance and continuation of behavior had a significant positive correlation with Emotional Transformation, Practice for Change, and Changes in Social Environment, three influencing components based on the MTM model (P < 0.0001).

**Table 5:** Results of a stepwise multiple regression model predicting the smoking cessation initiation in participant samples.

Predictors	Unstandardized coefficients		Standardized coefficients	t	*p*-value
	B	SE	Beta		
(Constant)	0.754	0.198	–	3.804	<0.001
Participatory dialog	0.002	0.018	0.008	0.109	0.913
Behavioral confidence	0.054	0.024	0.232	2.290	0.024
Changes in physical environment	0.149	0.043	0.355	3.500	0.001
R = 0.547 R^2^ = 0.299 ADJ. R^2^ = 0.289					

**Table 6:** Results of a stepwise multiple regression model predicting the sustenance of smoking cessation behavior in participant samples.

Predictors	Unstandardized coefficients		Standardized coefficients	t	*p-*value
	B	SE	Beta		
(Constant)	0.170	0.255		0.664	0.508
Emotional transformation	0.117	0.034	0.301	3.403	0.001
Practice for change	0.096	0.034	0.247	2.802	0.006
Changes in social environment	0.080	0.031	0.188	2.604	0.010
R = 0.580 R^2^ = 0.337 ADJ. R^2^ = 0.323					

## Discussion

This study was conducted to examine if the MTM constructs can predict both the intention to initiate smoking behavior and the intention to maintain it among the male health workers in the west of Kabul City.

Many studies have so far been conducted on smoking behavior based on MTM, including smoking cessation, tobacco cessation, water pipe smoking, cigarette smoking, and substance use behavior (16, 19–22, 37–39). The initiation and sustenance of behavior change were substantiated in predicting health behavior changes by the findings of these studies. An average of good intention was reported for initiation (i.e., quitting in the near future) and sustainability of smoking cessation (i.e., sustaining smoking cessation for the foreseeable future) among the participants.

There was an increase in the R^2^ value of both the initiation and sustenance regression models after addition of the MTM constructs. The behavioral confidence of smokers and changes in physical environment were significant predictors of initiation of smoking cessation behavior. Sustenance for smoking cessation behavior was significantly predicted by emotional transformation, practice for change and changes in social environment. Sumitra Sharma et al. reported that 48% of the variance in initiation of smoking cessation was explained by behavioral confidence and changes in physical environment (19). This study and the study conducted in Nepal showed that behavioral confidence and changes in physical environment are more effective in predicting the initiation of behavior change. However, the study conducted in the United States found that only behavioral confidence was effective in initiating behavior change. It is likely that the difference in the percentage of predicting smoking cessation initiation behavior in the United States (23.6%) vs. Nepal (48%) and Afghanistan (28.9%) could be related to differences between the study contexts. Additionally, a positive correlation was found between the MTM variables and the initiation of smoking cessation.

There has been a significant relationship between perceived behavioral control and self-efficacy and smoking cessation reported in the literature. Studies show that smoking cessation rates are higher with higher self-efficacy (10). Research suggests that perceived behavioral control and self-efficacy should be exerted in the effort to diminish smoking behavior, and behavioral modification needs to be emphasized as a tool to enhance the level of success in quitting smoking (10, 16, 40). Behavioral confidence is an important predictor of cigarette smoking cessation and water pipe smoking reduction (16, 19, 21); the present study also confirms these findings. Studies on the MTM constructs and other behavior-related health measures (e.g., physical activity, consumption of sugar-sweetened beverages, binge drinking, eating habits, sleep, outdoor nature contact behavior, and mammography screening) have shown that behavioral confidence has consistently been an important predictor of behavior change initiation (24, 25, 27–30, 32, 33, 40, 41).

According to our study, physical environment changes significantly contributed to smoking cessation. it means that by removing all cigarettes from the environment, we can be able to refrain from purchasing cigarettes; this is a concept derived from Bandura’s social cognitive theory, referred to as *substitutability of activities* in substituting smoking time with something else (16, 21, 23, 24, 26, 29, 32, 33, 35–40, 42–45). Changing the physical environment was found to be an important predictor of a person’s decision to quit smoking in our study, which is consistent with the study of smoking cessation in Kathmandu Metropolitan City, Nepal (19). Initiation of other health behaviors is also predicted by MTM, including overdrinking, sugar-sweetened beverages, physical activity, outdoor nature contact behavior, SAVOR (Sisters Adding Fruits and Vegetables for Optimal Results), HPV vaccination, and mammography screenings (19, 25, 27, 28, 30–33, 40). Different health behaviors across countries are often influenced by environmental factors in combination with individual, family, and community factors (46).

Emotional transformation, practice for change and Changes in social environment were found to be significant predictors in the sustenance model, which explained 32.3% of the variance. Similarly, Nahar et al. reported that only emotional transformation was a significant predictor that explained 23.3% of the variance (16). Sumitra Sharma et al. (2020) found that 54% of the variance was explained by emotional transformation (19). In these three studies, emotional transformation played a key role in maintaining behavior change; again, this role is more prominent in Nepal and Afghanistan than in the United States. This reveals that maintaining behavior change is as effective as initiating behavior. Sustenance is positively correlated with three variables. Emotional intelligence is essential to the transformation of an individual’s emotions, and consequently, can be used in MTM to direct emotion in a way that will help the smokers quit and change their behavior by overcoming uncertainty (37). There is a positive correlation between emotional transformation and the sustainability of studies on smoking cessation (16, 19), tobacco cessation (20), water pipe smoking (21), smoking cessation in youth (22) and vaping quitting behavior (47).

In our study, smoking cessation sustainment was significantly predicted by practice for change. This predictor has been identified as a significant predictor of behavior sustenance in previous studies exploring MTM constructs like cigarette smoking and water pipe smoking in youth (21, 22). MTM has also been found to be associated with the practice for change and sustainability of other health behaviors in previous studies utilizing the MTM constructs such as outdoor nature contact behavior, SAVOR, physical activity, vaccination, sugar-sweetened beverages, and mammography screening (27, 28, 30–33).

Smoking cessation sustenance was significantly predicted by changes in social environment in the present study. An investigation by Abasi et al., titled “Cigarette smoking in youth, using MTM,” revealed that social environment plays a significant role in the sustenance of cigarette smoking among youth (20), which is consistent with our study. Using MTM to examine the social environment, other health behaviors were found to be significantly influenced by the social environment, including binge drinking, sleep, physical activity, dietary behavior, outdoor nature contact behavior, SAVOR, and mammography screening (24, 25, 28–30, 33, 40). Furthermore, other studies have indicated positive effects on smoking cessation behavior in the form of family and peer support (33, 48).

### Strengths and limitations

The application of the new health behavior change theory (MTM) is a key strength of this study, as it offers a unique approach for understanding and influencing health behaviors. However, the authors would like to point out that this study has some limitations. Due to the restrictions in the target population, a convenience sampling method was employed for male health worker smokers. As a result, the findings may not be applicable to populations beyond the sample used in this study. Furthermore, future research should consider employing randomized controlled designs to assess the effectiveness of interventions. The current study was limited to male health worker smokers, and future studies could explore both genders for a more comprehensive understanding. Lastly, our sample was drawn from the western part of Kabul City, meaning it does not fully represent all districts, which may influence the generalizability of the results.

### Future direction

Considering the limitation of the current study design, future research should prioritize the use of randomized controlled trials (RCTs) to evaluate the effectiveness of interventions. These trials could provide stronger evidence of the predictive capacity of the MTM constructs in various settings. Furthermore, health workers that include participants from both genders and diverse regions would contribute to a more comprehensive understanding of smoking cessation and behavior change across a wider population.

### Implications for future interventions

The results of this study can guide future interventions aimed at smoking cessation, particularly for male health workers. The MTM constructs, especially behavioral confidence and changes in the physical and social environments, should be further explored in developing customized interventions to improve smoking cessation outcomes. Additionally, further research could examine how these constructs interact with other psychological factors and external influences within diverse populations.

### Contextual differences

It is important to recognize that the findings of this study may differ from those of similar studies conducted in other countries, such as the United States or Nepal, due to contextual variations. For instance, cultural norms, social support systems, and the availability of smoking cessation resources can all influence the effectiveness of smoking cessation interventions. Future research should investigate these contextual factors to gain a deeper understanding of how the MTM constructs function across different environments.

## Conclusion

Health workers in the west of Kabul City showed significant predictor relationships with two MTM constructs for initiation change and three MTM constructs for maintenance change. The MTM was shown to be accurate at both predicting the initiation of smoking cessation and its sustainability. In this study, MTM constructs were successful in initiating and maintaining behavior change through the development of smoking cessation interventions. Based on the MTM model, behavioral confidence and changes in physical environment constructs were significant for starting smoking cessation, and emotional transformation, practice for change and changes in social environment constructs, were significant for maintaining behavior change. For the development of tobacco cessation interventions in the future, more studies are needed to determine whether the MTM constructs are predictive in other more diverse and randomized samples to improve generalizability.

## Data Availability

The original contributions presented in the study are included in the article/supplementary material, further inquiries can be directed to the corresponding author.
